# Supplementary material for: Quinazoline Derivative kzl052 Suppresses Prostate Cancer by Targeting WRN Helicase to Stabilize DNA Replication Forks
Source: Int J Mol Sci. 2025 Jun 25;26(13):6093. doi: 10.3390/ijms26136093 (PMC12249509; doi:10.3390/ijms26136093)
Supplement: Supplementary file 1 [file ijms-26-06093-s001.zip › ijms-3632247-supplementary.pdf]

# Supplementary data

Figure S1.

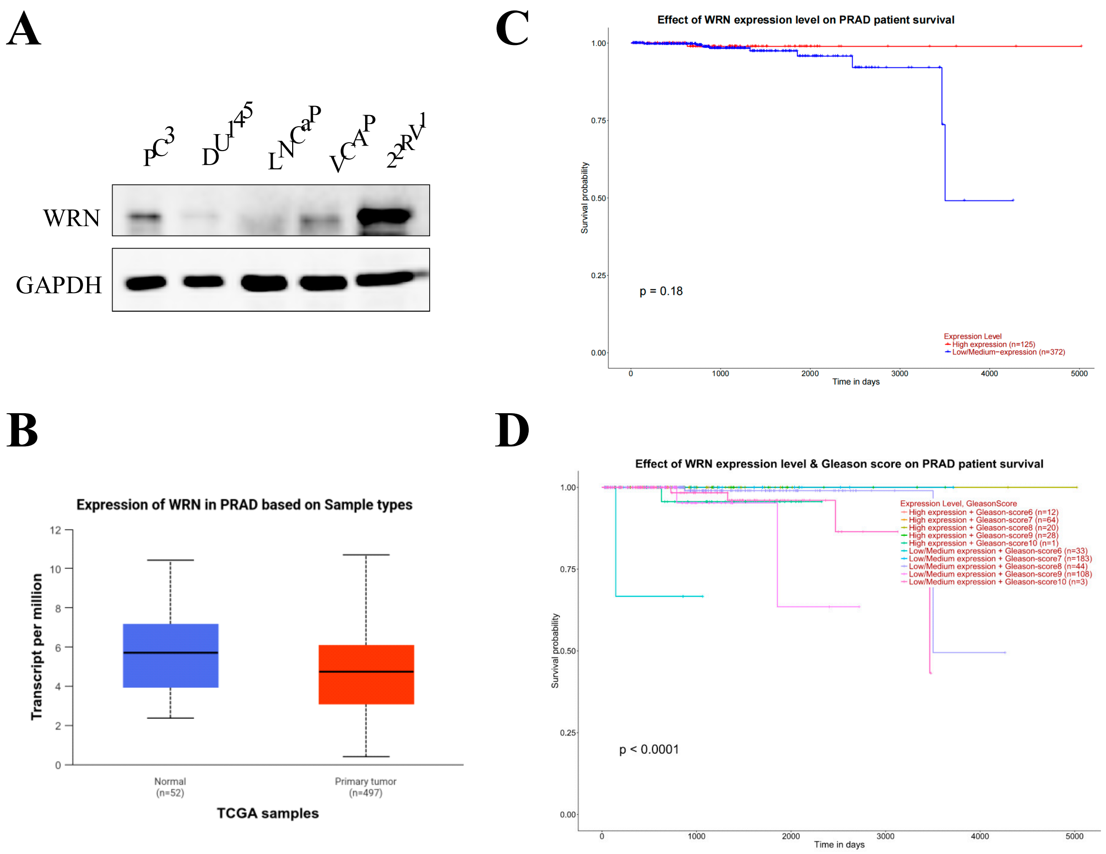


Figure S1. WRN in prostate cancer. (A) The expression level of WRN protein in different prostate cancer cell lines. (B) The WRN protein level in Clinical prostate cancer tissues and adjacent tissues. (C) The effect of WRN expression level on prostate cancer patient survival. (D) The correlation between WRN expression and different stages of prostate cancer.

Figure S2.

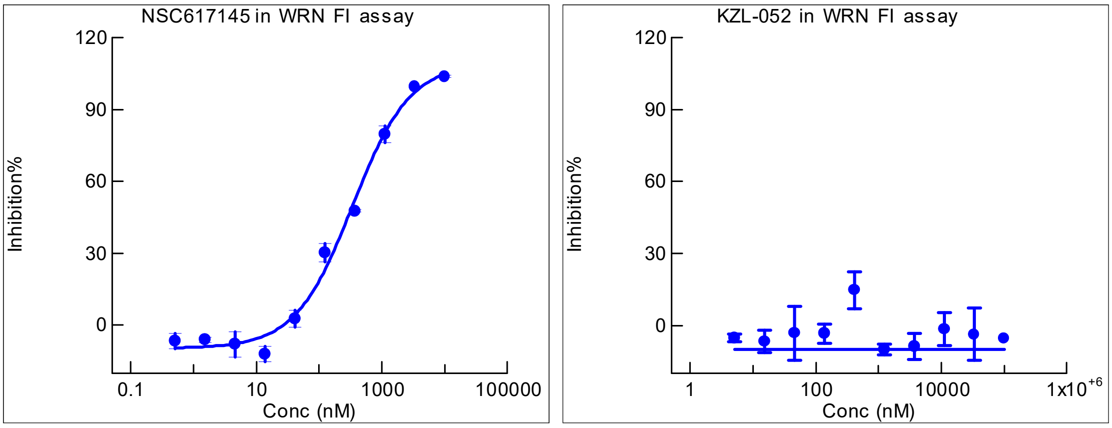


Figure S2.The results of WRN-unwinding assay. WRN enzyme activity test were performed by ICE Bioscience Inc (Beijing, China).
